# Diclazuril Protects against Maternal Gastrointestinal Syndrome and Congenital Toxoplasmosis

**DOI:** 10.4236/ijcm.2014.53017

**Published:** 2014-01-01

**Authors:** Helieh S. Oz, Thomas Tobin

**Affiliations:** 1 Department Internal Medicine, University of Kentucky Medical Center, Lexington, USA; 2 The Maxwell H. Gluck Equine Research Center, Department of Veterinary Science, College of Agriculture, University of Kentucky, Lexington, USA

**Keywords:** Diclazuril, Gastrointestinal, Hepatitis, Colitis, Pregnancy, Congenital Toxoplasmosis

## Abstract

**Background:**

Toxoplasmosis is a common cause of foodborne, gastrointestinal and congenital syndrome with particularly severe or unknown health consequences. There is no safe and effective preventive or therapeutic modality against congenital toxoplasmosis or to eliminate the persistent chronic infection.

**Hypothesis:**

Diclazuril to be safe in pregnancy and effective against gastrointestinal toxoplasmosis.

**Methods:**

CD1 programmed pregnant mice were divided into groups and administered a diet containing diclazuril, or sham control. Treatments were initiated on Day 5 of pregnancy and continued until Day 16 when dams were euthanatized. On Day 8 of pregnancy dams were infected intraperitoneally with escalating doses of tachyzoites (0, 100, 300, 600) from Type II strain. Dams were monitored daily for distress, pain, and abortion and samples collected at the end of the experiments.

**Results:**

Infected dams developed moderate to severe *Toxoplasma* related complications in tachyzoites dose dependent manner. Animals became anemic and showed hydrothorax, and ascities. Diclazuril effectively protected dams from ascities and anemia (p < 0.05). Infected dams showed splenomegaly, with massive infiltration of epithelioid cells compared with the protective effect of diclazuril in treated animals. Infected dams exhibited severe hepatitis (score 0 to 4 scale = 3.5 ± 0.01) with influx of inflammatory and plasma cells, dysplastic hepatocytes, multinucleated giant cell transformation and hepatic cells necrosis. Diclazuril treatment significantly protected dams from hepatitis, also in tachyzoites dose (100, 300, 600) dependent manner (respectively infected-treated versus infected controls, p < 0.001, p < 0.01 and p < 0.05). Colonic tissues were significantly shortened in length, with infiltration of lymphocytes, and macrophages and microabscess formations in the cryptic structures, with significant improvement in diclazuril treated animals. Additionally, the number of fetuses, fetal length and fetal weight were preserved in diclazuril treated dams.

**Conclusions:**

This is the first report describing of diclazuril safety in pregnancy as well as efficacy against mild to moderate hepato-gastrointestinal syndrome in dams and fetal toxoplasmosis (Special issue, “Treatment of Liver Diseases”).

## 1. Introduction

*Toxoplasma gondii* is an important source of foodborne hospitalization and congenital disorders that infects humans and animals. Toxoplasmosis manifests with gastrointestinal disorders to severe cerebral, ocular and fetal complications. Toxoplasmosis in immunocompetent people is typically symptomless or may appear as flu like syndrome. However, it can lead to severe complications and death in immunocompromised hosts, fetus and neonates [1]. It is predicted that 1,500,000 cases of toxoplasmosis occur in the USA each year, and about 15% of these cases reveal clinical symptoms [2].

Latent toxoplasmosis can become reactivated mainly in AIDS and in immunocompromised patients and during pregnancy. Congenital toxoplasmosis occurs in man and animals [3] by transplacental transmission of organisms during maternal infection. Maternal-fetal transmission was first reported in an infant in New York in 1939 [4], and its consequences have long been recognized [5]. Congenital toxoplasmosis manifests with spontaneous abortion, intrauterine fetal death or severe congenital defects, including encephalitis [6].

There is currently no safe and effective (FDA approved) therapy against congenital toxoplasmosis to prevent or eliminate fetal complications and persistent chronic *Toxoplasma* infections. Current therapies include spiramycin alone or associated with pyrimethamine-sulfadoxine, to prevent transfer of *Toxoplasma* from the actively infected mother to the fetus and to treat the infected fetus. However, this approach is not always effective and is associated with unwanted side effects [7–10]. Atovaquone (hydroxy-1,4-naphthoquinone) which is an FDA approved treatment for toxoplasmosis yet is not in use for congenital toxoplasmosis [7]. Recently, we have reported that atovaquone protects dams and their fetuses against some infectious and inflammatory aspects of toxoplasmosis [11].

Diclazuril [4-chlorophenyl [2,6-dichloro-4-(4,5-dihydro-3H-3,5-dioxo-1,2,4-triazin-2-yl)pheny l acetonitrile] is widely used in prevention and treatment of poultry and livestock coccidiosis and Equine Protozoal Myeloencephalitis (EPM) in horses [12]. Diclazuril is a safe compound [13] with no known side effects at therapeutic dose levels. However, diclazuril has not been tested or used in pregnancy.

Diclazuril acts by specifically targeting a chloroplast derived chlorophyll a-D1 complex present in Apicomplexan and not represented in mammalian systems [14]. As such, diclazuril and its related substances have the potential for high anti-Apicomplexan therapeutic efficacy and minimal mammalian toxicity. Diclazuril has not been tested in pregnancy in general or congenital toxoplasmosis.

The objectives of this study were diclazuril to be safe in pregnancy and effective treatment against the maternal gastrointestinal syndrome as well as progression of congenital toxoplasmosis in our murine pregnancy model of toxoplasmosis.

## 2. Materials and Methods

### 2.1. Ethics

This research was conducted according to the guidelines and approved by the Institutional Biosaftey Committee (IBC) and the IACUC at University of Kentucky Medical Center.

### 2.2. *Toxoplasma gondii* Propagation

*Toxoplasma* Type II isolates including ME-49 strain are predominant in human congenital toxoplasmosis [15]. For this investigation, *Toxoplasma* organisms from PTG strain (ME-49, ATCC50841™) were originally cloned, and propagated by Dr. Daniel Howe from Maxwell H. Gluck Equine Center at the University of Kentucky [11, 16]. Briefly, tachyzoites were cultured by serial passage in bovine turbinate cells and maintained in minimum essential Medium (MEM-RS, HyClone Labs, Inc.) supplemented with 4% fetal clone III (HyClone, Labs, Inc.), Penicillin/Streptomycin/Fungizone (BioWhittaker, Inc.), and non-essential amino acids solution (HyClone, Labs, Inc.). Upon disruption of the host cell monolayer, extra-cellular tachyzoites were harvested and purified from host cell debris by filtration through 3.0 μm membranes. Tachyzoites were enumerated in a hemocytometer and suspended in phosphate buffer saline (PBS) to the appropriate concentrations for inoculation. All inoculations were administered intraperitoneally (*i.p*.) in 100 μl volume into dams within 1h of harvest to ensure viability.

### 2.3. Murine Fetal Toxoplasmosis Model

Murine play an important role in propagation and transmission of *Toxoplasma* and are commonly used in studying toxoplasmosis. In addition, CD1 mice are hardy animals with relatively large number of pups and routinely used in pregnancy investigations (11, 17). Therefore, sixty Day 1 programmed pregnant (9 weeks old) CD1 dams were purchased from Charles River Lab Inc. Wilmington, MA). Dams were housed individually in micro-isolator cages in a pathogen free environment and maintained at 22 °C with a 12:12-hr light: dark cycle at the Maxwell H. Gluck Equine Research Center Laboratory Animal Facility. Dams were fed irradiated rodent chow and sterilized drinking water *ad libitum*. After acclimation, (Day 8 pregnancy) dams were weighed and ear-punched for appropriate identification. They were assigned into 6 – 10 animals per group, and injected *i.p*. with 100 μl PBS containing 0, 100, 300, or 600 tachyzoites using 0.5 ml insulin syringes. Control dams received 100 μl sham injection with PBS alone. Animals were monitored 3 times daily for physical appearance, distress, pain, diarrhea and vaginal discharge to detect early delivery or abortion [11,17]. The experiment was terminated on gestation Day 16 before possible early or premature birth to study the feto-maternal aspects of the disease.

Animals were euthanatized using CO_2_ inhalation. Their chests were immediately opened and blood from heart collected in microtainer (BD Biosource, Rockville, MD) for hematocrit evaluation. Sera were separated and stored frozen at −80 °C. The splenic weight and length were recorded. Heart, liver and uterus were excised and weighed. Colonic contents were removed and colonic length and weight recorded then flash frozen in liquid nitrogen and stored at −80 °C for future studies. Live fetuses were removed from uteri, counted, weighed and their lengths measured using a digital caliper.

### 2.4. Diclazuril Treatment

Diclazuril is currently used in coccidiosis in poultry and EPM in equines but has not been tested in pregnancy or congenital toxoplasmosis. In order to study safety and efficacy of 65 mg/kg and 120 mg/kg diclazuril against fetal and maternal toxoplasmosis complications, animals were divided into groups of 6 – 8 animals. Dams received treatment regimens incorporated into daily diet as indicated in our previous publication [11]. Control groups received sham treatment (inert talcum powder). Treatment was initiated on Day 5 of pregnancy and continued until Day 16 when dams were euthanatized. Pregnant animals’ were monitored daily for food consumption and changes in their appearance, food consumption or weight loss/gain. On Day 8 of pregnancy dams on treatment or sham control arms were further divided into 4 subgroups of 6 – 8 animals each and were injected each with 0 (PBS), 100, 300, or 600 tachyzoites and the treatments continued. Three different tachyzoites infective doses were chosen based on our previous study [11] to develop a mild (100), moderate (300) and severe (600) toxoplasmosis to best represent the common disease process in infected patients in general. This range also enabled us to perform the investigation in accordance with IACUC regulation to prevent excess animals use and unnecessary demise.

### 2.5. Histopathological Examination

#### 2.5.1. Hepatic Tissues

A portion of the right lobe from liver tissues was placed in cassettes and fixed with 10% neutral formalin. The specimens were dehydrated and embedded in paraffin, and tissue sections of 5 μm were stained by Hematoxylin Eosin (H & E) [11,18]. Each slide was evaluated under Ziess light microscopy. Hepatic lesions were graded on a scale of 0 to 4+, based on degeneration, inflammation, and necrosis as follows:

Grade 0: No detectable lesions, no degeneration, no infiltration of inflammatory cells, normal tissue appearance.
Grade 1: Focal infiltration of inflammatory cells in the hepatic structure, and random hepatocytes degeneration.
Grade 2: Mild multi-focal infiltration of inflammatory cells, and hepatocytes degeneration.
Grade 3: Moderate multi-focal infiltration of inflammatory cells, and hepatocytes degeneration.
Grade 4: Severe diffuse inflammation, and necrosis.

#### 2.5.2. Colonic Tissue and H & E Staining

Colonic tissues were flushed with PBS (pH 7.2) and a portion from proximal and distal colonic tissue was fixed in 10% neutral formalin for histological examinations. The remainder was snap-frozen in liquid nitrogen and stored at −80 °C. The formalin fixed sections were processed and stained with hematoxylin and eosin (H & E), and slides evaluated by Ziess light microscopy. Severity of colitis was assessed with a histological semi-quantitative grading score and performed in a blinded fashion. The scores were based on histopathological features with a numeric value (0-normal to 4-severe) assigned according to the tissue involvement [19,20] that corresponded to either of the following criteria:

Grade 0: No detectable lesions, no inflammatory cells, normal mucosal appearance.
Grade 1: Focal inflammatory infiltrate in the mucosa.
Grade 2: Mild multi-focal inflammation with moderate expansion of the mucosa.
Grade 3: Moderate multi-focal inflammation with moderate expansion of the mucosa.
Grade 4: Severe diffuse inflammation with crypt epithelium disruption and ulceration.

#### 2.5.3. Giemsa Staining

Giemsa is a delicate polychromatic stain that reveals a fine nuclear detail of *Toxoplasma* organisms. Giemsa stain contains Methylene Blue Azure Basic dyes combined with Eosin Acidic dyes. The deparaffinized slide sections were stained with the polychromatic Giemsa (40 drops/50 ml distilled water) to stain nuclei of the *Toxoplasma* organisms and to permit differentiation among the cells. Then, the slides were depreciated in 1% Glacial Acetic Acid, dehydrated in alcohol and xylene series and mounted in synthetic resin on slides.

#### 2.5.4. Immunohistochemical Staining (IHC)

Anti-*Toxoplasma* antibody and IHC procedure were kindly provided by Dr David S. Lindsay at at Department of Biomedical Science and Pathology, Virginia Tech. Briefly, paraffin-embedded sections were cut, deparaffinized with xylene, rehydrated in alcohol baths, washed in PBS with 0.1% BSA, The endogenous peroxidase activity was quenched by incubating in 3% hydrogen peroxide in methanol for 30 min, then blocked with rabbit serum (Dako #N1699), 30 min. The sections were incubated with polyclonal Rh anti-*Toxoplasma* antibody, diluted 1:500 for 90 min and developed with DAB-Chromogen (Dako Carpenteria, CA) for about 5 min until signal developed and subsequently counterstained with hematoxylin then ammonia treated and dehydrated stepwise through alcohol, clear with xylene.

### 2.6. Behavioral Test: Assessment of Pain Related Mechanical Allodynia by Testing Abdominal Withdrawal Threshold

Abdominal withdrawal responses to mechanical stimuli were quantified with von Frey monofilaments (Semmes-Weinstein Anesthesiometer Kit) according to our previous publications with some modification [11,21]. Briefly, mice were placed into plastic enclosures on the custom made screen meshed platform. The monofilament range used for this study included 5 different intensities corresponding to (hair diameter) gram force [(4.08) 1.0 g; (3.61) 0.4 g; (3.22) 0.166 g; (2.83) 0.07; (2.36) 0.02 g forces]. Testing for mechanical stimulation was performed on the first and the last days of treatment. A single trial consisted of 5 applications of the each filament used once every 6 seconds to allow the dam to cease any response and return to an inactive position. Mean values of the percentage of responses of the abdominal withdrawal to each filament (mean withdrawal/5 × 100) were used as % scores in this study. This behavioral test reflected basal level for reflex score and any possible sensory changes observed in the treated mice. Total 4 dams were tested per each group.

### 2.7. Statistical Analysis

Results are expressed as mean ± SEM unless otherwise stated. Data was evaluated with ANOVA followed by appropriate *post hoc* test (Tukey compared all pairs) using GraphPad Instat version 3 for Windows (GraphPad Software, San Diego, CA). Statistical significance was set at p < 0.05.

## 3. Results

### 3.1. Drug Safety and Tolerance

In the initial study, naïve dams were provided diets containing two different doses of low (65 mg/kg) and high (120 mg/kg) diclazuril or sham treatment. All dams voluntarily consumed their diets with no adverse effect of clinical symptoms, changes in body weight gain rates or appearance detected in diclazuril treated dams compared to those receiving the sham treatment.

### 3.2. Diclazuril Dose Related Efficacy and Toxoplasmosis in Model

We compared efficacy of low and high doses of diclazuril in dams infected with 0 or 100 tachyzoites during the 2^nd^ trimester pregnancy. Infected dams demonstrated moderate complications related to toxoplasmosis. As expected sham treatment provided no protective changes on the severity of the disease; while, dams treated with high dose diclazuril were significantly more protected against *Toxoplasma* infection and clinical symptoms compared to those receiving the low dose of diclazuril treatments (data not shown). Therefore, we selected the high dose (120 mg/kg) diclazuril for the following investigations.

### 3.3. Efficacy of Diclazuril against Severity of Toxoplasmosis

In the next study, we compared efficacy of high dose diclazuril (120 mg/kg) in dams infected with different quantity of single inoculum (0, 100, 300, 600) tachyzoites. Infected dams developed moderate to severe toxoplasmosis complications in tachyzoites dose dependent manner. Dams infected with high dose tachyzoites exhibited significant increases in body weight mainly due to edema (p < 0.01) (Table 1). Additionally, these dams became anemic (Controls 44.5 ± 1.2 versus infected-dams 37 ± 2 p < 0.05) and developed hydrothorax and ascities. Diclazuril treatment effectively protected dams from anemia (41.1 ± 2.4), and excess pathological weight gain as a result of ascities (Table 1).

### 3.4. Splenic Pathology

**Figure 1:**
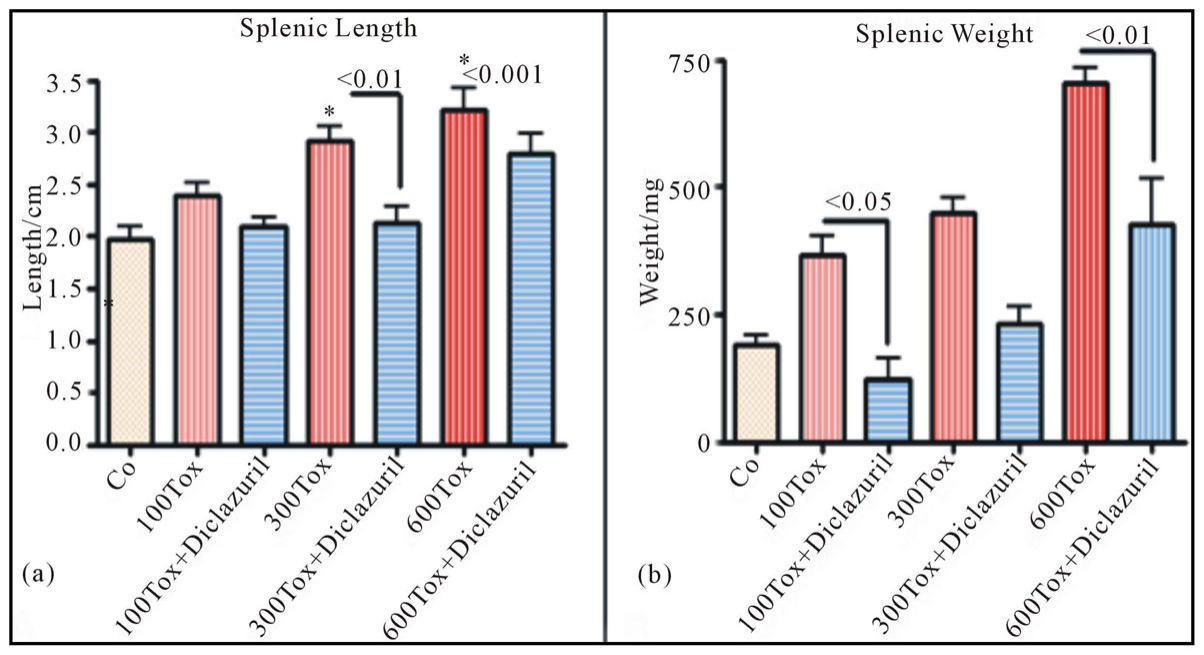
Splenic length (a) and weight (b) increased due to inflammatory response in infected dams in tachyzoites dose dependent manner and diclazuril significantly protected dams from pathogenesis. (a) = Splenic length from Control (Co) or infected with 300 tachyzoites (300 Tox)-diclazuril treated vs infected dams p < 0.01; Control vs infected with 600 tachyzoites (600 Tox) p *<* 0.001 (n = 6/group).

Infected dams demonstrated splenomegaly in tachyzoites dose related manner (2 – 3 fold of normal control values), with massive infiltration of epithelioid cells and loss of germinal structure with significant increases in weight and length of splenic tissues compared with significant protection in infected but diclazuril treated dams (Figures 1(a) and (b)).

### 3.5. Hepatic Pathology

**Figure 2:**
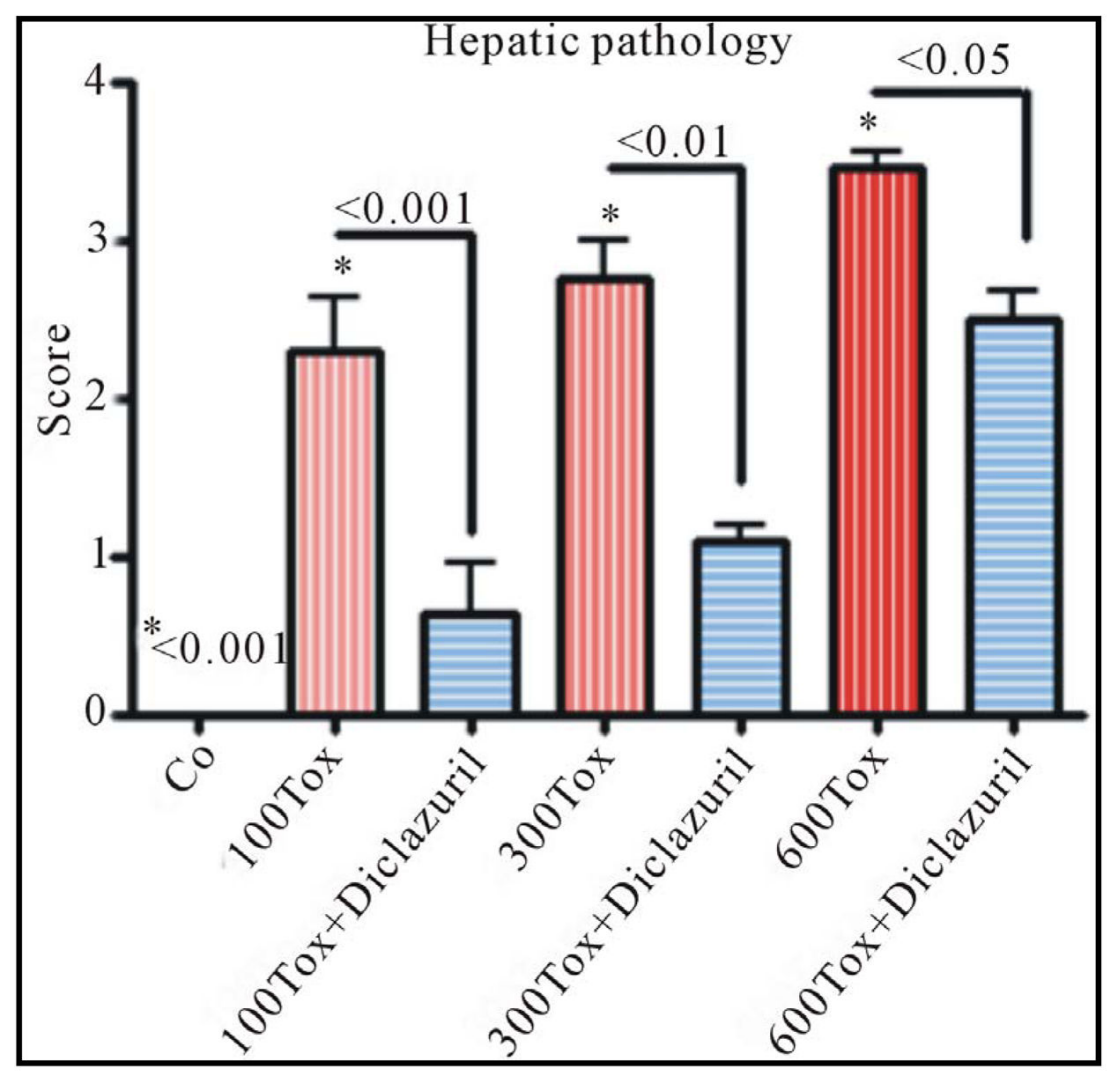
Slides from hepatic sections stained with H &E and scored from 0 normal control (Co) to 4 (most severe lesions) for pathological findings from infected dams compared with those treated with diclazuril. Hepatic pathology scores increased in tachyzoites (Tox) dose dependent manner and diclazuril significantly protected the animals from hepatic injuries.

Hepatic tissue became pale in appearance and increased in weight due to infection and inflammatory response. Infected dams illustrated a mild to severe hepatitis [pathology score on scale 0 – 4 most severe lesions: 2.3 ± 0.4 (100), 2.8 ± 0.3 (300), and 3.5 ± 0.01 (600 tachyzoites)] with distorted hepatic architecture, dysplastic hepatocytes formation, and multinucleated giant cells transformation. In addition, moderate to diffused influx of inflammatory cells, including polymorphonuclear, mononuclear and plasma cells, depicted in parenchyma with hepatocytes degeneration and necrosis in tachyzoites dose depended manner. Scattered tachyzoites in parenchyma were confirmed with Giemsa staining. Diclazuril treatment significantly protected dams from hepatic injury also in tachyzoites dose related manner (100, 300, 600) and accordingly ameliorated the hepatic pathology (infected-diclazuril treated versus infected sham treated p < 0.001, p < 0.01 and p < 0.05, Figure 2).

### 3.6. Cardiac Pathology

Dams exhibited mild to moderate myocarditis with infiltration of inflammatory cells into cardiomyocyte tissue, embedding scattered pseudocysts and tachyzoites (Table 1). This was consistent with the detection of no pseudocysts in the cardiac tissues from diclazuril treated animals. The presence of the scattered tachyzoites or pseudocysts was confirmed with, Giemsa and IHC staining of the tissue sections (not shown). Diclazuril treated dams were all protected and did not develop any detectable *Toxoplasma* organisms.

### 3.7. Fetal Numbers, Weight and Length

**Figure 3:**
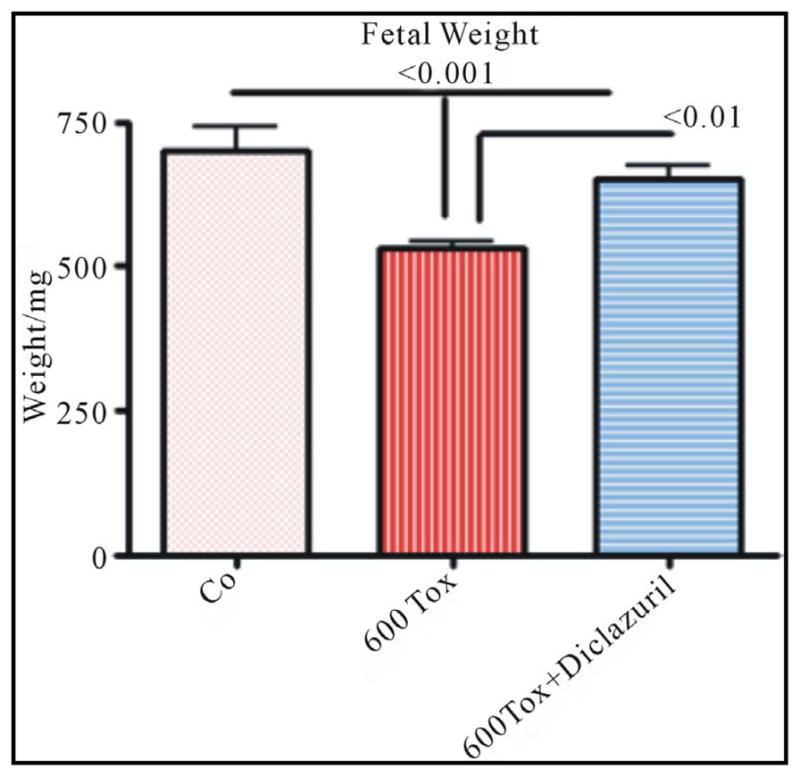
Fetal weight from control (Co) dams, infected with tachyzoites (Tox) compared with those treated with diclazuril.

Most importantly, the fetal weight and length and the number of fetuses were significantly affected in infected dams with high dose tachyzoites (600 tachyzoites) leading to fetal retardation and/or fetal loss. In contrast the number of fetuses was protected in infected and diclazuril treated dams and the fetal length and fetal weights were significantly improved (Figure 3).

### 3.8. Pain Related Abdominal Response to Stimuli and Diclazuril Therapy

Infected dams (600 tachyzoites) showed a significant pain related abdominal hypersensitivity (*p* < 0.01) to von Fray mechanical stimuli as demonstrated with abdominal spasm and withdrawal compared to the uninfected controls. However, diclazuril treatment had no significant improvement on the abdominal hypersensitivity in infected dams as compared to infected and sham treated animals (Table 1).

## 4. Discussion

Numerous reports indicate the clinical importance of toxoplasmosis complications and the need for a safe and effective therapeutic specifically for chronic infection and congenital toxoplasmosis [22]. The objectives of this investigation were to evaluate the efficacy of diclazuril against the progression of the gastrointestinal syndrome and maternal fetal toxoplasmosis in our murine model.

*Toxoplasma*, a Category B classification by CDC and NIH, is a common parasite of animals, and causes severe foodborne diseases and in fetal, neonate and immuno-compromised (AIDS/HIV) patients. Congenital transmission of toxoplasmosis may result in serious complications including fetal death or lifelong adverse consequences [1,23,24].

Present therapies for toxoplasmosis include pyrimethamine, sulfasalazine, sulfadiazine and spiramycin, which these therapies are not always effective and/or have a major side effects. Pyrimethamine is a pregnancy category C drug and can cause severe side effects including bone marrow suppression in both mother as well as infant. In addition, more than half (58%) patients who receive spiramycin retain *Toxoplasma* DNA in their peripheral blood [9]. Although, a case of fetal bradycardia has been claimed to be associated with spiramycin therapy [10] in general, spiramcyin is well tolerated and is more of preventive in early pregnancy but not efficacious when parasite has already passed the placenta.

In a 20 year prospective clinical trial (1985–2005) of infected mothers (666/676) who were treated with spiramycin alone or combined with pyrimethamine-sulfadoxine with live born children, 112 (17%) confirmed with congenital toxoplasmosis and, 26% later developed chorioretinitis [8]. Similarly, in another study in France, from 257 live births from women with prenatal positive diagnosis for the toxoplasmosis were treated with both spiramycin and pyrimethamine-sulfadoxine, 66 (24%) children were infected, yielding transmission rates of toxoplasmosis of 7% in the first, 24% in the second, and 59% in the third trimesters, respectively [25].

Given these circumstances, there is a great need for more effective therapeutic modalities with less toxicity and better tolerated regimens for fetal and congenital toxoplasmosis as well as for the recurrent *Toxoplasma* related disease.

In this report we have demonstrated that diclazuril treatment significantly reduces pathogenesis in maternal and fetal infection with toxoplasmosis during pregnancy. This is the first report demonstrating diclazuril to be safe and effective in pregnant dams as well as their nested fetuses and in higher doses than currently used in prevention of coccidiosis.

Diclazuril is mainly used in poultry industry to protect against coccidal infection and to treat clinical cases of EPM infection in horses caused by Apicomplexan protozoa *S. neurona* that is closely related to *Toxoplasma* [12,26–28]. Previously, diclazuril in lower dose (10 mg/kg) was shown to be effective against experimental *Toxoplasma* infection in male mice [29] and an infected Hawaiian (Alala) bird [30].

Diclazuril [4-chlorophenyl [2,6-dichloro-4-(4,5-dihydro-3H-3,5-dioxo-1,2,4-triazin-2-yl)pheny l acetonitrile], is a benzeneacetonitrile compound [26,27]. Following oral administration, it is rapidly absorbed to achieve steady-state concentration in plasma and cerebrospinal fluid (CSF) with peak concentrations occurring at 8 – 24 h after single dose, sufficient to interfere with *S. neurona* proliferation with 95% inhibition rate [26,27].

A major therapeutic advantage for dicalzuril is its highly specific anti-Apicomplexan effect because Apicomplexan agents, including *Toxoplasma*, are unusual among protozoa in that they contain protochlorophyllidae with traces of chlorophyll bound to the photosynthetic reaction centers [14]. The diclazuril/herbicide-binding region is shown to be highly conserved in Apicomplexans, which results in a unique sensitivity of Apicomplexans to the herbicide related drug, diclazuril and associated benzene acetonitriles. Therefore, diclazuril binds the chloroplast epitope and interacts with the D1 protein of the photosynthetic reaction center of *Toxoplasma* organelles without damaging the mammalian host cells. The fact that mammalian systems lack the protochlorophyllidae related diclazuril/herbicide binding region results in a very high therapeutic index for diclazuril and related agents. In addition, diclazuril is shown to down-regulate expression of serine/threonine protein phosphatase (EtRACK) in second-generation merozoite of *Eimeria tenella* to induce apoptosis [31]. EtRACK has about 98% homology with *Toxoplasma* that demonstrates a possible mechanism of action for diclazuril. Recently, we reported efficacy of atovaquone (hydroxy-1,4-naphthoquinone) against some aspects of gastrointestinal complications in congenital toxoplasmosis [11]. However, diclazuril reveals better efficacy against anemia, colonic length improvement, hepatic complications and excess body weight. On the other hand, atovaquone was more effective in reducing pain related abdominal response to mechanical stimuli [11]. Therefore, diclazuril treatment protected dams against mild to moderate gastrointestinal syndrome and their fetuses from *Toxoplasma* infection.

## 5. Conclusion

This is the first report describing of diclazuril safety in pregnancy and efficacy against mild to moderate hepato-gastrointestinal complication and congenital toxoplasmosis for which no satisfactory therapy is currently available. As such, these findings warrant further investigations of this novel prophylactic and therapeutic approach to gastrointestinal as well as congenital toxoplasmosis.

## Figures and Tables

**Table 1:** Efficacy of diclazuril on infected dams in fetomaternal toxoplasmosis model.

Tissues	Control	Infected	Infected + treated
Excess body weight (%)	0	62 ± 1^a^	41 ± 2.5^a^
Hematocrit (%)	44.5 ± 1.2	37 ± 1.4^a^	41.1 ± 2.4
Hepatic weight (g)	1.78 ± 0.25^b^	3.4 ± 0.14^b,a^	3.18 ± 0.99^a^
Colonic length (mm)	125 ± 2.5	87 ± 2.2^a^	99 ± 4^a^
Heart weight (mg)	202 ± 10	315 ± 30^a^	247 ± 18^a^
Uterus weight (g)	11.60 ± 0.5^a^	13.9 ± 0.7^a^	11.9 ± 1.9
Hypersensitivity (%)	20 ± 0.5^b^	43 ± 2.5	40 ± 0.4

Tissues from normal control dams (**Control**), those infected with 600 tachyzoites from PTG strain, treated with sham (**infected**), and those Diclazuril treated dams (**Infected + treated**). Animals were monitored daily X3/until Day 16 of pregnancy before termination. Abdominal hypersensitivity to von Fray stimuli increased significantly in infected animals. p *value* ^b^ < 0.01; ^a^ < 0.05.

## Abbreviation

BSA: Bovine serum albumin
CDC: Center for Disease Control
CNS: Central nervous system
EPM: Equine Protozoal Myeloencephalitis
FDA: Food and Drug administration
H & E: Hematoxylin Eosin
IACUC: Institutional Animal Care Use Committee
IBC: Institutional Biosaftey Committee
IHC: Immunohistochemical staining
*i.p*: Intraperitoneally
MEM: Minimum essential medium
PBS: phosphate buffer saline
*S. neurona*: *Sarcocystis neurona*
